# Examining NEET situations in Spain: Labour Market, Discourses and Policies

**DOI:** 10.1007/s43151-021-00048-2

**Published:** 2021-08-10

**Authors:** Tanja Strecker, Joffre López, M. Àngels Cabasés

**Affiliations:** 1 Departament de Geografia i Sociologia, Universitat de Lleida, Lleida, Catalonia Spain; 2 Departament d’Economia Aplicada, Universitat de Lleida, Lleida, Catalonia Spain; 3 Spanish Youth Council, Madrid, Spain

**Keywords:** NEET, Youth Guarantee, Spain, Catalonia, Youth policies, Labour market

## Abstract

Not in Education, Employment, or Training (NEET) and its Spanish equivalent ‘nini’ (Ni estudia, Ni trabaja) have dominated youth policy discourses in recent years. Within the European Union, Spain is one of the countries with the highest proportion of young people in NEET situations. In this article, it is argued that the idea of NEET has been weaponised to stigmatise youth, by evoking the phantom of a demotivated young person with scarce training. This stigmatisation has little to do with the reality of many young Spaniards who can find themselves in different situations, such as unemployment, precarious employment, training and education in a matter of days. Thus, there is a need to consider the different experiences and structural circumstances of so-called NEETs rather than viewing them as a homogenous and static group. Using documentary analysis and secondary data, this article examines the diversity of NEET situations for the youth in Spain, which is generally not captured in large national statistics data-sets and policies. Furthermore, it analyses the EU Youth Guarantee and its application in Spain, highlighting where official objectives have not been met, and includes an overview of the current effects of the coronavirus crisis. Ultimately, the paper shows that public discourses centred on an artificially created social group (NEET) legitimise and produce policies that do not respond to young people’s actual needs and problems, especially for the most vulnerable among them.

## Introduction

The notion of Not in Education, Employment, or Training (NEET) and its Spanish equivalent ‘nini’ (Ni estudia, Ni trabaja) have been buzzwords in public and policy discourses in recent years. According to Eurostat data (2020), Spain is one of the countries with the highest proportion of young people in NEET situations. As a consequence of the economic and social crisis of 2008, the situation of young people in the European and Spanish labour market (Furlong 2013) has further deteriorated, including high unemployment rates, job instability and low salaries; which had already become common characteristics of youth employment before (Moreno 2012a, b; Furlong 2013; Alonso et al. 2016; Cabasés Piqué et al. 2016). Such labour deterioration leaves young people in a vulnerable position when new crises — like the current coronavirus pandemic — arrive. This scenario makes it relevant to analyse why youth policies fail to address the issue of NEET situations and youth labour precarity. The aim of this article is to contribute to the closure of this gap.

In this article, official statistics and secondary data are analysed to depict the current youth labour market and its impact on contemporary transitions to adulthood. Conceptual and epistemological reflections on the ‘NEET’/‘nini’ concept, based on the analysis of political discourse, public policies and scholarly literature, are provided, and various public policies are also considered in this analysis. A threefold approach that analyses the contemporary situation of youth labour is provided, together with a conceptual discussion of the NEET/nini notions and discourse, and the analysis of youth and employment policies, with a focus on the EU Youth Guarantee. Ultimately, this threefold approach reveals that the current model of youth employment is marked by precariousness and that the Youth Guarantee has not contributed to improve the situation of young people. On the contrary, its specific application in Spain furthers a model that intensifies the already highly precarious situation faced by young people in Spain.

## The Labour Market and the Youth Population in Spain: An Ambivalent Relationship

The chances of a young person being able to complete a positive transition to adulthood depends, among other things (e.g. access to the housing market, educational opportunities), on their incorporation into the labour market, including a certain degree of stability and employment on a full-time basis. This pattern, which is common in the European Union, is even more accentuated in Spain, where the differences in the employment situation between young people who have managed to leave the family home and those who remain in it have widened over the past years (Spanish Youth Council, 2020). It is mostly young people who are working on a full-time basis and with permanent contracts who are able to become independent from their family (Spanish Youth Council, 2020). In other words, the characteristics of the labour market have a direct impact on the options of a young person to afford to live independently. Nevertheless, currently, it cannot be assumed that all young people living at the parental home are absolutely dependent. As the internal relations within the families change, often new ‘family agreements’ (Gil Calvo 2002), new spaces of autonomy and new dynamics in economic and service transfers between family members emerge (Gil Calvo 2002). The role played by family ties, housing policy and even the social construction of the concept of ‘youth’ need to be taken into account when considering issues of autonomy.

In strictly labour terms, young people in Spain are currently distinguished by a dual dimension in comparison to their European peers: firstly, their weaker position in the labour market; secondly, their higher risk of finding themselves in precarious jobs (temporary works, involuntary part-time jobs, low-skilled jobs, no options to promote, low earnings, discontinuous jobs, irregular...)^1^. Until 2013, the activity rate^2^ of young people in Spain exceeded that of every country in the European Union. Since then, it has been progressively declining, and by the second quarter of 2020, only Italy, Serbia, Greece, Bulgaria and Montenegro recorded lower percentages than Spain (44.6%) (EUROSTAT 2020a, b)^3^. The socio-economic crisis caused by the COVID-19 pandemic has further accentuated this ‘disappearance’ of young people from the labour market in Spain. Even among the young population ‘inside’ the labour market, another differential factor emerges in Spain: the incidence of unemployment. Since the start of the last real estate fall down, in late 2007 and early 2008 (Rodríguez, 2017), the youth unemployment rate had increased exponentially to 44.3% in the first quarter of 2013. At that time, the unemployment rate for the entire young population (15–29 years) of the European Union was less than half this figure (20.5%). After its peak in 2013, unemployment gradually decreased among the youth population in Spain, not so much due to job creation, but rather because of the transition by young people from unemployment to ‘non-active’ status (López 2020). This continued until 2020 when, for the first time since 2013, unemployment increased again in the first two quarters of the year. In the second quarter of 2020, no country in the European Union had a higher youth unemployment rate than Spain, which was at 30.0% while the average in the European Union was 12.9% (EUROSTAT 2020a, c).

The second characteristic that distinguishes the incorporation of young people into the labour market is their precariousness. It is a phenomenon exclusive neither to Spain (Caddy 2015) nor to the young population; however, among young people in Spain, job insecurity acquires a particular meaning. Precariousness translates into multiple situations that feed each other; for example, contractual temporality, involuntary part-time hours, underemployment, overqualification, the proliferation of intermittent periods between unemployment and employment, low expectations of personal advancement, informal work (and, consequently, not protected or recognised by public administrations) and, obviously, reduced wages (Spanish Economic and Social Council 2020). The problem is aggravated for young people who when they lose their job, they often do not meet the minimum requirements to receive unemployment benefit. In many countries, unemployment benefits work as a basic safety net against the risk of poverty (see Furlong et al., 2018; Viera et al. 2018; Cuervo and Chesters 2019). In a recent report, however, the Economic and Social Council ( 2020) stated that:

Economic protection against the risk of poverty derived from unemployment has a limited scope (measured through the coverage rate) and is further reduced for the young population, given their difficulties in finding employment for the first time and the characteristics of the labour market, such as high turnover and intermittent employment. The lower protective effect on the young population is striking, even more so, when considering that unemployment protection is one of the few functions of social protection in which Spanish expenditure is above the European Union average (Spanish Economic and Social Council 2020, p. 149).

Following the socio-economic crisis resulting from the COVID-19 pandemic, inequalities in employment and working conditions have become even more accentuated for those in a vulnerable position. They have been the first to experience the effects of the cessation of non-essential activities and the increase in unemployment. As with the 2008 crisis, employment in the public sector has become a refuge from economic collapse, even for the young people, who constitute a small percentage of the population employed in some public administrations. In the second semester of 2020, only 10.4% of the salaried population aged 16 to 29 in Spain was working in the public sector, compared to 26.6% of the rest of the population (Spanish Youth Council, 2020).

Although young people constituted the age group with the highest rate of job losses in 2020, this experience cannot be attributed solely to their age; that is, one can also find differences within youth as a generational group. For example, young people with permanent contracts have had a better chance of continuing in their job than people with temporary contracts which, on many occasions, have not been renewed. During the pandemic, young workers in non-permanent contract or those who held jobs that require little academic training (such as, for example, the so-called ‘elementary jobs’) lost their jobs at a higher rate than those working on a permanent contract status (Spanish Youth Council, 2020). This does not mean that those who managed to keep their jobs left the current crisis unscathed. Rather, a phenomenon shaking up the job market today is the proliferation of Temporary Workforce Reduction Schemes (ERTE furloughs), through which companies can temporarily lay-off their workers, who receive a benefit to partially cover their salaries^4^ and keep up their Social Security contributions. Since the population affected by ERTEs is not technically unemployed, it is still a working population. In the absence of data broken down by age, one has to resort to the Labour Force Survey (LFS) to estimate the scope of ERTEs and find out how many people have not worked at all due to employment regulation records or partial unemployment for technical or economic reasons. The results in 2020 show that in the second quarter almost 30% of the employed young population had their work activity suspended. Among the non-youth population, the extent was also notable, though lower (23.8%) (López 2020).

Once more, young people cannot be considered a homogeneous block when it comes to evaluating the impact of ERTEs. It has been young people with the most unstable and precarious jobs who have been more likely to be furloughed. For example, compared to 29.6% of the entire young population, the suspension of working hours due to workforce reduction schemes or partial unemployment has affected 32.1% of young women and 34.0% of foreign nationals. In terms of education, 33.6% of the population that has completed compulsory secondary education and 34.0% of those who have completed post-compulsory secondary education were affected. The most affected sector was the industry sector with more than 60% of the population employed in the hotel industry or in activities related to the arts and entertainment, and 46.6% of those who work in restaurants and commerce.^5^ Therefore, it should come as no surprise, for instance, that the Balearic Islands and the Canary Islands have been the two Autonomous Communities where the workforce reduction schemes have had the greatest impact since their economic structure is significantly dependent on the tourism sector (Spanish Youth Council, 2020).

The ability to cushion the economic effects (and other effects that transcend the purely economic sphere) of the new macroeconomic scenario that has emerged from the COVID-19 pandemic depends on the situation previously enjoyed by young people and households. This, of course, reinforces inequalities over time. The effects of prolonged unemployment are particularly significant for young people entering the labour market, as they might neither be preferred by employers later when the economic situation has improved and new graduates and young labour market entrees are available, turning them into a ‘lost generation’ that never achieves stable employment (Furlong 2013, p.79).

## Conceptualisations Around the Notions of NEET and Nini

According to Eurostat, Spain can be distinguished as a country with a high rate of young people in NEET situations^6^. In the Spanish context, the equivalent to NEET is nini (*ni estudiando, ni trabajando* — neither studying nor working): a concept that was first coined in a trade union report to denounce the maladjustment between education and the labour market and the growing exclusion of young people from both (UGT 2005). The aim of the trade union was to change public policies to favour the labour position of young people (Feixa and Strecker 2016). Similarly, the English equivalent NEET was designed to be a less ‘discriminating’ term compared to ‘inactive’ and ‘Status Zero’ (Vancea and Utzet 2018, p. 2) and to include young people who are not registered as unemployed but are still neither working, nor studying (Furlong 2013). However, both concepts rather quickly became buzzwords, mostly used to stigmatise young people (Cabasés Piqué et al. 2016; Yates and Payne 2006). The focus is on what young people are missing out on, indicating a lack of motivation on their part, rather than considering the difficult and precarious context they are facing. NEET or nini definitions also include judgemental add-ons that automatically relate to all young people who are neither studying nor working. Vallejo (2017, p. 221) calculates that, of the 21% of young people considered ninis in Spain, only 0.5% ‘state having no interest in seeking employment’ — a particularly small group when compared to the high number of young people who are both studying and working. Salvà-Mut et al. (2018, p. 8) found that almost all NEETs in Spain are ‘available and looking for employment’ and that those who are available but not looking for employment have ‘a history of greater labour participation and job instability’. Conceptualisations of NEET, however, portray young people as “a group ‘without qualities’”, with their ‘employability’ needing to be improved, whereas often ‘social problems are turned into individual deficits’ (Serrano Pascual and Martín Martín 2017, p. 1). The idea is activating youth and employment policies that aim to adapt young people to the labour market rather than creating work opportunities and promoting a structural change in the employment market (Assusa 2019; Cabasés Piqué et al. 2016; Lahusen et al. 2013). Moreover, in Spain, activation has been combined with particularly ‘harsh austerity policies’ (Rodriguez-Modroño 2019, p. 13), marked by severe cut-backs in public expenditure across the board. Amnesty International (2018), for instance, has come to denounce some of these measures, for violating the right to health. Other researchers show that the effects of these measures have been further economic deprivation and the increase of social inequality (Picatoste 2017).

Various studies have discussed the complexities and heterogeneities of ninis and criticised the ‘social disqualification’ that this term implies, but continue to build upon ‘suppositions, worries and classifications’ based on ‘the political-communicational construction of the problem’ rather than a sociological approach (Assusa 2019, p. 92). According to Assusa (2019, p. 107), the category nini neither corresponds to “‘effectively existing’ statistical groups”, nor to ‘homogenous social conditions’, that is to say ‘ninis are neither young, unemployed, criminals, a new problem, nor an enduring social condition’. However, the use of the category enables ‘different types of social agents to construct *generational and social class others*’, accused of the ‘worst moral vices of current western societies: laziness, unproductivity, lack of autonomy and violence’ (Assusa 2019, p. 107, italics in the original).

For Vallejo Peña (2017), young people are used as a scapegoat to divert attention from the mistakes and deficits of the Spanish institutions when explaining the difficult situation they are facing. This is particularly worrying in the context of the growing precarity shown in the previous section.

The question is, therefore, who is neglected through the use of the label NEET/nini? The focus on ninis in public discourse firstly makes young people who are in employment and education but experience precarity (Furlong 2013) invisible. In Spain, the concept *mileurista* initially referred to a highly educated and trained young person earning €1000 or less per month, and hence being unable to become independent (Feixa and Strecker 2016). The focus here is on the maladjustment of the salary of highly educated young people, questioning the usefulness of higher education, while low salaries for people with little or no education are not criticised (Vallejo Peña 2017). In the wake of the crisis of 2008, many young people experienced lower earning power, while precarity continued to increase, especially for young women (Úbeda et al. 2020). By focusing public and policy discourse on ninis, attention is drawn away from these precarisation processes, and little or no help is offered to young people in this situation. Stigma is, indeed, also affecting people facing poverty, even while working, as in many situations in daily life where poverty becomes obvious, onlookers know nothing about the labour situation of the affected person, and so that even educated workers can come to feel stigma and its terrible effects for their well-being and health (Tyler 2020).

Furthermore, the concept nini itself also diverts attention from young people who fall into this broad category, which includes young people on a sabbatical, preparing for the selection process for public employment, caring for children or parents, ill or with a disability or without a valid work permit (Furlong 2013; MacDonald 2011; Salvà-Mut et al. 2018; Serracant 2012; Strecker and Cabasés 2019; Mascherini et al. 2012). For many young — and not so young — people, rapid changes between different statuses are increasingly common. A person can be on a training course one day, unemployed the next, completing an unpaid internship the day after, returning to unemployment after a matter of days, and enrolling for another training course and so on, and all this without even considering informal labour situations. Continuous change (Madsen et al. 2013; Vancea and Utzet 2018) and ‘chaotic transitions’ (MacDonald 2011, p. 8) are in this sense far more common than static situations of long-term inactivity. The nini label creates the impression of a static homogenous group, concealing how many young people enter and leave NEET situations within a brief period of time.

Moreover, among the young people who are neither working nor studying and who are ready and legally able to accept work offers, different profiles should be distinguished: it is by no means the same to be a young university graduate, seeking temporary employment while waiting for a placement in their field of study (Strecker 2019), than to live in a situation of high vulnerability, without any educational qualifications or recognised work experience (Strecker and Cabasés 2019). Although the category of NEET is broad, the risk of falling into a NEET situation depends on ‘gender, educational level, age, origin, place of residence, socio-economic background and health’ (Vancea and Utzet 2018, p. 3) and is ‘highest for those with poor educational attainments, with a low household income, with a disability or with those who are immigrants’ (Furlong 2013, p.81). In order to offer the attention and support needed, these different profiles should be distinguished, rather than including all in the broad category of NEET.

Similarly, social class, educational level and family support are decisive in ‘mitigating the impact of the first contact with the labour market’ (Vallejo Peña 2017, p. 221); that is, precarity is not only unequally distributed, its effects are also very different for young people from different social strata, further increasing social inequalities (Antonucci 2018; Cuervo and Chesters 2019). Further, Vancea and Utzet (2018) argue that young people from a poorer family background and/or who belong to a milieu of high unemployment (particularly among their generational peers) are more likely to be in a NEET situation and to have experienced a greater degree of unemployment than their counterparts. Similarly, ‘having a low level of education, being an immigrant, having an uneducated parent, having children at an early age, belonging to an economically deprived household, and a precarious state of health due to some sort of addiction are aspects that increase the likelihood of being NEET’ (Salvà-Mut et al. 2018, p. 10). Furthermore, age makes a difference, as young people aged 18 to 24 have different profiles from those aged 25 to 35, and other age groups (Vancea and Utzet 2018). A general focus on NEET, an ‘anti-ethical youth representation’ (Serrano Pascual and Martín Martín 2017, p. 1) leads to one-size-fits-all recipes that ignore gender, country of origin, social class and other structural differences.

By merging many different profiles into one category, public policies may artificially improve their success rates, as more young beneficiaries find their place in the labour market. Considering that they are artificially improved leaves the low success rates of youth employment policies, like the EU Youth Guarantee, in an even worse light. One could (and should) wonder the following: what would these rates look like if they focused on young people with the greatest difficulties to enter the labour market? How many of the young people who were working when the follow-up was carried out had found employment thanks to their participation in a public policy measure? How sustainable is their labour market integration and do they face precariousness? As is shown in the next section, the data available on recent youth employment initiatives like the EU Youth Guarantee is insufficient to answer these questions. Considering these issues with NEET/nini terminology and conceptualisations: why are NEET and nini so broadly used and readily accepted and adopted by public discourse? Part of the answer is the view on youth. Young people in Spain — and internationally — are viewed in public policy discourse as a problem: they only emerge in an *adult-centred* society, when there is a concern that they might be a risk to the social order by deviating from social norms (e.g. teenage pregnancy, drug use, extreme right-wing political orientations or issues with labour market integration) (see Duarte Quapper 2015; Wood 2017). It is usually out of concern with keeping the social order that resources are invested on their research and support and the focus is placed on their deficits; that is, the skills and resources they are lacking and their behaviours and attitudes deemed undesirable. This is often accompanied by ‘significant amounts of sensationalism in the media’ (Vallejo Peña 2017, p. 211), with young people criticised for being ‘frivolous, spoiled and consumerist’ (ibid., p. 216), blaming them for their unemployment situation and the precarity they encounter in a weak and unstable labour market. An artificial contrast is thus created: young people are treated as a homogeneous group in contrast to another homogenous group: adults.

The long tradition of such a deficit perspective has emerged once again in the current COVID-19 pandemic. According to a recent analysis (RAY-COR 2020), most research on the effect of COVID-19 on young people focused on worrisome elements like mental health issues and labour precariousness. Following the end of the lockdown period, young people were infantilised and scolded for spreading SARS COV2 with their *botellones* (binge drinking in public places) (Zafra 2020). Feixa qualified the Spanish confinement measures to reduce infection as marked by a ‘hygienist ideology’, an ‘excess of moralisation’ and an infantilisation of young people, who are observed with distrust and told how to behave and what to do with their bodies (Sen 2020). Moreover, Feixa believes that late independence from family — a result of the precarious labour market situation (Vallejo Peña 2017) — explains the worse infection rates in Spain than in other European countries, where young people move out earlier and live in flats with their peers, therefore being less likely to infect elderly family members (Sen 2020).

Youth employment policies in Spain and in the EU in general have long been criticised for ‘focusing too much on targets, indicators and expectations and too little on young people themselves’ (Feixa and Strecker 2016, p. 73); for adopting a ‘fire-fighting approach to working with young people rather than focusing support and intervention on areas where they may be most productive’ (Yates and Payne 2006, p. 329); and for a ‘cruel optimism’ in their focus on employability, given that without the creation of new jobs for qualified employees, higher levels of education only favour increased competition for the existing jobs (Bessant et al. 2017; Bessant and Watts 2014). This is especially true for young women in Spain who are at a higher risk of being in a NEET situation than male graduates despite holding a university degree (Rodriguez-Modroño 2019). The problem is that education is still put forward as solution panacea, allowing public policies to focus on educational rather than other structural measures to create employment and facilitate labour market integration. The deficit perspective applied to young people becomes particularly visible when these two subgroups are considered: young people with little or no education are told that they should study (or have studied) more (De Luca et al. 2020), and young people with education are told that they are too ‘fussy’ and should accept job offers even if they are below their educational levels and aspirations (Vallejo Peña 2017).

## Youth and Employment Policies and the EU Youth Guarantee

The commitment of the European Union (EU) to combat unemployment, especially youth unemployment, must be placed within the framework of the EU competences established in the Treaty on the Functioning of the European Union (TFEU). It should be remembered that the competence in employment corresponds to the nation-states, who must observe the general guidelines for economic policies and make them compatible with employment policies (Cabasés and Pardell 2014).

From 2010 onwards, the European Union intensified its actions directed at tackling the European unemployment rates caused by the 2008 economic crisis by putting forward a set of initiatives which led to the Council Recommendation of 22 April 2013 on the establishment of the Youth Guarantee. In Spain, it was implemented through the Youth Guarantee Implementation Plan (YGIP) and later with the Youth Guarantee System implementation plan (YGS) (Law 18/2014 and Royal Decree-Law 6/2016).

The YGIP focused on the NEET population — from young people who had prematurely abandoned their studies and possessed neither qualifications nor work experience, to university graduates with a range of skills and even prior work experience who were unsuccessfully searching for jobs — without considering all the variables that would have been included in a more holistic approach. It should be noted that the aforementioned Plan was initially oriented towards all NEETs under 25; however, this age limit was increased in 2015 to 29. That is, during the first 2 years, the Plan was extended to a wider age range than the one set by the EU (youths aged 16 to 24), covering young people up to age 30.

However, the YGS was not implemented until October 2014 through Act 18/2014, which established the requirements that young people had to meet in order to receive programme benefits. In other words, the YGS did not come into effect until a year after the Plan was passed.

Several evaluations of its implementation have revealed that the goals set by the European Recommendations (EC 2018a, b; ECA 2015, 2017) have not been met, leading to further frustration of active employment policies directed towards young people (Cabasés and Pardell 2014). In order to address how the YG did not comply with the objectives of the European Recommendation in Spain, the Youth Guarantee (YG) objectives are reviewed: to reach all young people in a NEET situation, to offer them a ‘good quality’ job or training and the offer should be made within a 4-month period. According to the State Public Employment Service (SPES) and the Labour Force Survey (LFS), in 2018, after 5 years of YG implementation, there were 1,083,336 young people in a NEET situation in Spain, 399,216 less than in 2014, while for 2018 the number of enrolments was 343,142, thus covering 31.7% of the possible beneficiaries.

Regarding the quality of the job offers, data shows that the YG has not been a guarantee of employment. The collective of young people in 2018, compared to other age groups, presented the highest unemployment rate (in spite of its reduction since 2014), the highest temporariness and the highest percentage of people underemployed; that is, working too few hours (Strecker and Cabasés 2019).

With regards to the maximum 4-month period between enrolling and receiving a job offer, an important aspect of the YG, it can be said that it has also not been totally accomplished.

Different European evaluations have revealed that a high percentage of young people who were beneficiaries of the YG returned to their NEET situation. In particular, the European Commission, in its 2018 report (EC 2018a, b) noted that half of the beneficiaries in 2016 and 2017 never left the YG circuit. The YG has not changed the immediate needs of young people, rather it has contributed to extending their waiting time towards complete immersion into the ‘adult world’, as well as rendering different situations of precariousness and vulnerability chronic (Úbeda and Sànchez 2018).

Most of the actions foreseen by the YGS were well designed, as they consider the level of education of the young beneficiaries of the YG, their level of professional qualification and their need to acquire certain competencies and skills. However, their design lacked an integral approach, as it failed to consider aspects of young people’s lives beyond employment, that is, intertwined mechanisms of social inequality and multiple exclusion, such as gender, ethnicity, age or social class, which led to the YG being called into question (Úbeda et al. 2020).

Furthermore, it was found that young people of international origin and/or belonging to ethnic minorities, who live in households in which no member works or who live in single-parent households, and young women with certain disadvantaged profiles, such as being inactive, with a low level of education, a disability, of immigrant origin, belonging to ethnic minorities or in a situation of housing deprivation, were underrepresented in the design of measures in the YGS. All in all, the European Recommendation has not been fulfilled (Ministry of Labour and Social Economy 2018).

The European Commission, in its report of February 2019 (EC 2019), asserted that subsidies for hiring within the YG framework had had limited success and had failed to promote quality employment. In addition, the report states that young workers with permanent subsidised contracts had more chances of losing their jobs within 2 years than those working under non-subsidised contracts, thus putting into question the efficacy of these subsidies.

The same report also called into question the quality of job offers that young people received within the framework of the YG, as they were a reflection of the situation of the labour market, thus challenging the impact of the YG on the labour market.

## New Solutions to Solve Perennial Problems? The Youth Employment Emergency Action Plan

In 2018, the Youth Employment Emergency Action Plan 2019–2021 (YEEAP) was approved to improve the management of the YGs. It contains a set of measures and actions aimed at improving the quality of employment, redressing the gender gap in labour, and reducing youth unemployment. In 2020, the background and the starting point analysed by the YEEAP changed. It was designed within a reality that has rapidly transformed during the COVID-19 pandemic, with little idea of where the youth labour market is heading. In fact, the evaluation that was due to take place after 18 months has not happened, and the set of indicators that was supposed to be established has also not been established yet, although in November 2019 the YEEAP monitoring committees were set up. Nevertheless, some considerations can be made regarding the YEEAP.

Firstly, the YEEAP addresses workers under the age of 30 with no employment or training, with the aim of ‘promoting their incorporation into the labour market, with good quality and stable jobs’. With this aim, it establishes the boosting mechanisms for actions contained in the YGs. It is worth noting that, while the YG addresses young people in a NEET situation, which includes inactive and unemployed people, the YEEAP, as stated in its Declaration of Commitment, reduces the target group of young people compared to the YGs.

Secondly, the YEEAP emphasises that the measures should ‘allow to cater for the individuality of the different groups of young people, who are obviously not a uniform collective’ and should ‘have a holistic and integral approach, be specific and yet combinable’ (YEEAP, p, 17). However, one major concern is that the measures have not been designed from an intersectional perspective. The measures contained in axis 1 — orientation —, address the whole youth collective and they focus on setting up the ORIENTAJOVEN programme. This establishes a network of 3000 people responsible for the orientation and prospection of employment services, and 110 mediators of the SIJ-INJUVE Network to provide synergies with professionals from other institutions to achieve a one-stop-shop effect for young people. Regarding the measures in axis 2 — training —, training programmes address young people registered as unemployed: in key competences for those who left education early; in digital competences where digital divide is a higher risk; in foreign language competences and second opportunity programmes for young people who have not completed their compulsory secondary education, especially the younger ones. The measures in axis 3 — employment opportunities — have the aim of boosting hiring, creating employment and maintaining jobs, placing special attention on young people in a situation of social exclusion. In general, these are booster measures and proposals of a regulatory nature, along the same lines as those in axis 4 — equal opportunities in accessing employment — and those in axis 5 — entrepreneurship.

Third, the YEEAP states that the set of measures it contains does not involve an increase in expenditure, as it will be funded by ‘the budget lines allocated in the statement of expenditure of the State Employment Service and the Ministry of Science and Innovation’ (YEEAP, p. 52). This is contradicted by the second agreement of the Resolution that establishes the Emergency Action Plan that reads, ‘All commitments derived from the implementation of this Plan are subject to existing budget availability for budget periods 2019, 2020 and 2021, in accordance with the fiscal consolidation established by the Government’. The question, therefore, is to what extent is this Plan a priority and an Emergency Action Plan, as its name would claim.

Finally, the YEEAP reduces the YG to a simple management tool and not a global system, as it had been envisaged in the first place — which included programmes and measures, the management tools to access all young people in a NEET situation and the evaluation of the implementation of the YG. Law 18/2014 laid the foundations for the YGS, taking the European Recommendation into account. This system had been defined as ‘consisting of support measures’ in the YGIP, and was to adjust to ‘the national, regional and local circumstances’. Specifically, Law 18/2014, set the following YGS goals: (a) that all young people in a NEET situation would receive a job, education or training opportunity, including apprenticeships or traineeships, after completing formal education or becoming unemployed; (b) develop support measures and programmes, and (c) monitor and evaluate all YG actions and programmes. Moreover, the YGS file was created as ‘the official information and monitoring system of the Youth Guarantee implementation’. Afterwards, Royal Decree-law 6/2016 was passed with the aim of continuing to boost the YGS through the adoption of measures directed at improving registration and assistance and fostering young people’s employability and employment. Ultimately, it sought to improve YGS management and efficacy. However, axis 6 of the Emergency Action Plan — improvement of the institutional framework — considers the YGS as ‘a basic tool for the development of the initiatives put forward within the Young Employment Plan’, and established as goal 1 ‘the improvement in YGS governance’ through eight measures.

In 2020, according to Decision (EU) 2020/1512 of the Council of 13 October, guideline number 6 reaffirms the EU’s commitment to the Youth Guarantee (YG): Youth unemployment and the issue of young people not in employment, education or training (NEETs) should continue to be addressed through the prevention of early school-leaving and the structural improvement of the school-to-work transition, including through the full implementation of the Youth Guarantee.

Moreover, it mentions the problems of youth unemployment and of young people in a NEET situation as two different problems, when they are actually linked, qualifying the NEET situation as an ‘issue’. Once again, the strategy to be adopted to solve the grave problem of youth unemployment is based on dubious categories such as NEET, without openly taking into account the heterogeneity and the dynamic changes between the different member states that make up the reality of today’s young people (Cabasés Piqué et al. 2016); and more so if considering the current serious situation derived from the COVID-19 pandemic and how it is exacerbating the problems of young people in the labour market, not only regarding their access to it, but also the precarious hiring conditions: temporality, part-time jobs, overqualification and low wages (Cabasés et al. 2017). Although this diagnosis regarding the special vulnerability of young people in the labour market has been socially shared (which had previously generated a great deal of measures and programmes over the years), paradoxically in the context of the crisis caused by the COVID-19 outbreak, it did not result in specific actions for the young population. Lockdown, benefits for the self-employed and furloughs were just universal measures.

It is also noted that the pandemic and periods of lockdown have a greater impact on more vulnerable economic sectors, with a higher presence of young people (López 2020). This is in addition to the greater risk young people face of losing their jobs when the lay-off ends, depending on the impact of COVID-19 on the economy. Finally, it is important to highlight that the young people who were in a precarious situation before the pandemic and those who were in a NEET situation may suffer the consequences more severely.

The measures in the YGIP, such as the most recent YEEAP, have not taken into account a thorough analysis of the situation faced by the young population from a comprehensive point of view that analyses these individuals’ ways of life as well as their difficulties. This has given rise to training programmes of little value, designed to justify EU investments, which fail to bring about greater stability in the lives of young people. As most of the measures have been based on the acquisition of competencies and individual skills, they leave the young person responsible for his or her precarious situation and vulnerability. Youth policies do not address structural problems, leading to the individualisation of social problems and risks.

## Conclusion

In this article, a threefold approach was taken to young people in a NEET situation in Spain: firstly, the contemporary situation of youth labour, followed by a discussion of the NEET/nini notions and discourse, and an analysis of youth and employment policies, with a focus on the EU Youth Guarantee.

The article shows how public discourse and policies continue to adopt a deficit perspective on young people whereby the focus is placed on the lack of skills and experience (París et al. 2006; Strecker and Cabasés 2019). The concept of NEET tends to stigmatise young people as lacking motivation and skills, rather than focusing on the structural challenges and inequalities they face. NEET/nini terminology contributes to the ‘invisibilisation’ of young people (Feixa and Strecker 2016), particularly the neglection of the high degree of heterogeneous profiles of young people in NEET situations and the precarisation processes inflicted on young labour market entrees. This invisibility or neglection of youth’s heterogenous composition and experiences is especially worrisome as it enables public policies to focus on ‘easy’ short-term remedies, for instance educational measures, rather than offering individualised support based on the concrete situation of each young person and investing in structural changes in the labour market, job creation and job quality guarantees for young people and the population at large. Ultimately, the tendencies outlined are in line with the concept of a moral *juvenicidio *(the symbolic and economic ‘assassination’ of young people) (Strecker et al. 2018), as young people are made invisibles and blamed for the economic hardship that is inflicted upon them.

As the different sections of this article have shown, it is important to critically analyse statistical data and public policies considering the discourses and concepts they relate to. From such analysis, it becomes clear that the EU Youth Guarantee is indirectly built on stigmatising concepts and discourses such as ‘NEET’ or ‘nini’. This may explain why the policies designed do not respond to young people’s actual needs and problems, failing to offer adequate strategies to support young people in their transitions. European and Spanish national policies often only act as short-term remedies and contribute to rendering NEET situations recurrent, instead of addressing the structural problems that cause them. Furthermore, the evaluation of their measures is deliberately misleading, as different profiles of young people are mixed in one category, failing to complete a systematic evaluation of the long- and medium-term impacts. This, in turn, has a negative effect on the available statistics and official data, making it difficult to analyse the realities of young people and the effectiveness of public policies in other terms.

All of the above taken together depicts a vicious circle in which current youth policies in Spain and the EU are trapped: Young people’s realities are observed from a negative standpoint, focusing on their deficits and ignoring structural causes and heterogeneous profiles. Public policies are built on this negative perspective, embodied in concepts like ‘NEET’, when it comes to youth labour, or ‘reckless super spreaders’, in the context of the current COVID-19 pandemic, to mention only two recent examples of this deficit discourse. Public policies are doomed to be ineffective if they do not broaden their perspective to take into consideration structural issues, heterogeneous profiles and young people as active citizens, who are ready to contribute (and already contribute) to a better future, rather than being the objects of victimising, ‘invisibilising’ and stigmatising policies.

## Footnotes

1 It is important to note that not all experiences of precarious work lead to global precarity because we must consider family resources and the welfare mix (Antonucci 2018).

2 The activity rate expresses the percentage of people who are actively working or seeking employment, an essential requirement for a person to be considered unemployed.

3 Authors’ elaboration from Eurostat data for the population aged 15 to 29.

4 With a maximum limit equivalent to unemployment benefit (70% of the basic salary for 180 days and 50% thereafter). <https://www.sepe.es/HomeSepe/Personas/distributiva-prestaciones/quierocobrar-el- paro/afectadoERE> [1 December 2020].

5 Source: Authors’ own from Labour Force Survey (LFS) microdata.

6 In Spain, in 2019, 14.9% of the total population aged between 15 and 29 years was in a NEET situation. The average for the European Union was 12.6%. <https://ec.europa.eu/eurostat/databrowser/view/EDAT_LFSE_20 custom_782413/default/table?lang=en>
